# Undercover Peripheral Arterial Disease

**DOI:** 10.7759/cureus.51590

**Published:** 2024-01-03

**Authors:** Catarina Costa, Beatriz Riquito, Sofia Perdigão, Rita Cunha, Victor Paz

**Affiliations:** 1 Internal Medicine, Centro Hospitalar Universitário do Algarve, Faro, PRT; 2 Internal Medicine, Centro Hospitalar de Trás-os-Montes e Alto Douro, Chaves, PRT

**Keywords:** cerebrovascular disease, polyvascular disease, stroke, multidisciplinary work, cardiovascular risk, atherosclerosis, peripheral arterial disease

## Abstract

Peripheral arterial disease is a frequently underdiagnosed disease that can severely affect the quality of life. We present a clinical case of a 62-year-old smoker post-menopause woman with a mild stroke. Further investigation revealed a severe disseminated arterial disease. Due to multidisciplinary and timely interventions, peripheral ischemia was prevented successfully.

In fact, this patient had polyvascular disease. Despite its worst prognosis than either coronary artery disease, cerebrovascular disease, or peripheral arterial disease alone, polyvascular disease is still underdiagnosed. Atherosclerosis and cardiovascular risk should be regarded as multisystemic and managed as such in multidisciplinary teams. A proper and timely intervention is essential to diminish its morbidity and mortality.

## Introduction

We know that vascular disease has high morbidity and mortality rates, mainly due to cardiac events [1]. This has led to a division of the vascular disease approach, with each medical specialty focusing on only one vascular area, instead of a global approach and screening. Not affecting vital organs, peripheral arterial disease (PAD) is usually managed by internists and general medicine physicians as a milder clinical problem, relegated to a second plan [2]. Due to delays, including delayed referral, patients' morbidity and risk of amputation are increased [3]. Understanding the multifactorial etiology and multiorgan involvement of atherosclerosis by all the physicians treating these patients is key to diminishing cardiovascular mortality and morbidity [3].

Here, we present a clinical case where cardiovascular risk was approached regardless of the presenting signs and symptoms. These allowed us to go further than the routine workup, in a multidisciplinary collaboration, with the internist as the main interplay, referring to the specialties needed.

This clinical case was previously presented as an e-poster at the 20th European Congress of Internal Medicine on June 9-11, 2022.

## Case presentation

**Figure 1:**
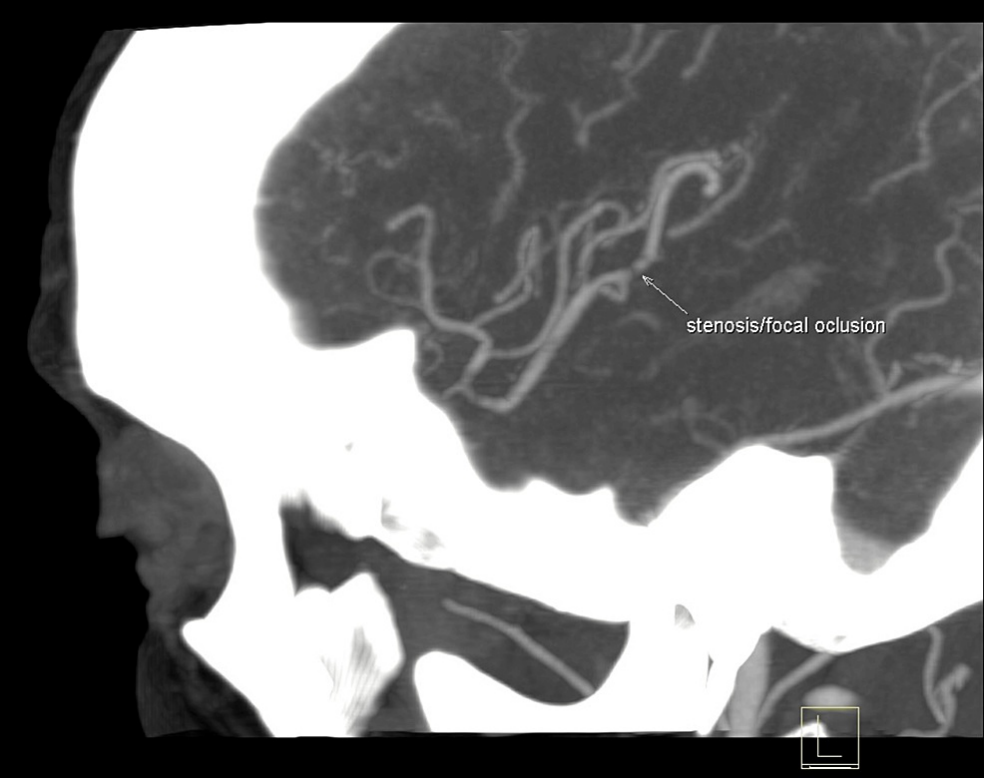
Head computed tomography with angiogram showing a focal occlusion at M2-M3 of the left medial cerebral artery

A 62-year-old right-handed woman with a medical history of active smoking habits and depressive syndrome is admitted to the emergency department for mild dysarthria, a discrete right hemiparesis (grade 4 out of 5), and a right central facial nerve palsy with 20 minutes of evolution. A head and neck computed tomography with an angiogram (CTA) was performed, showing no brain infarction but a focal occlusion at the left medial cerebral artery in M2-M3 (Figure 1), as well as atheromatous irregularities at the carotid bifurcation with no critical stenosis. Neurological deficits were improving since admission, with none of them compromising significantly her job, as a housekeeper, or her daily activities, and scoring 4 points on the National Institute of Health Stroke Scale (NIHSS). As so, she was not a candidate for thrombolysis. Also, due to the thrombus location, thrombectomy was not an option either. She was then admitted to the internal medicine ward for monitoring and further stroke workup, starting secondary prevention.

**Figure 2:**
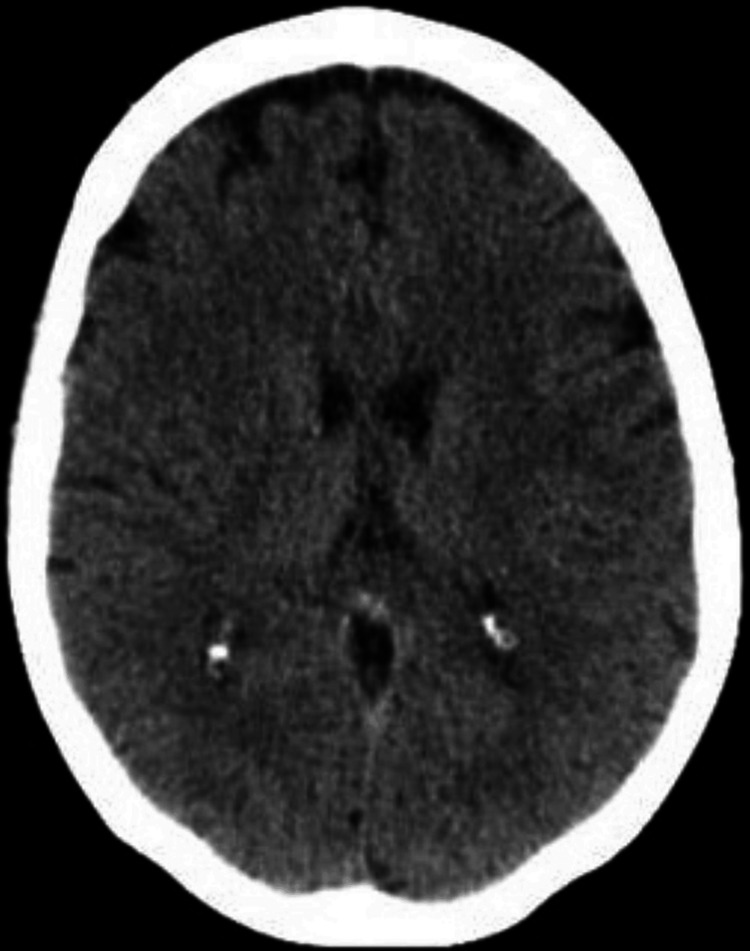
Head computed tomography showing small subcortical hypodensities

The patient was under continuous cardiac monitoring for 24 hours with no arrhythmias detected. Forty-eight hours after the event, no focal neurological deficits were observed. Since she was feeling better and after explaining the reasons for her admission, the patient chose to discharge herself on the fourth day of admission, allowing only a brief study as an inpatient. The short cardiovascular risk workup showed a hemoglobin A1c of 6.0% and a slightly high total cholesterol level of 193 mg/dL with high LDL levels of 120 mg/dL and low HDL levels of 25 mg/dL. Triglycerides were also slightly above the upper reference level (160 mg/dL). A detailed review of her past medical history was also made, discovering some transitory left hemianopsia seven months before. The head computed tomography (CT) was reviewed, showing small subcortical hypodensities, suggestive of chronic ischemia (Figure 2). No other relevant medical history was found apart from the depressive syndrome. She was discharged from the hospital medicated with atorvastatin 40 mg od and acetylsalicylic acid 150mg od and with indication of smoking cessation and ambulatory blood pressure monitoring. The patient's economic capacity and worry about statins' adverse effects were obstacles to the medication choice. She was referred to an internal medicine appointment to complete the remaining study.

After the first appointment, where several tests were ordered, the patient was found in the emergency department several times. The complaints varied from sharp foot pain to hand numbness and, more than once, asymptomatic low blood pressure (as ambulatory measurement), coincidently always measured on the left arm.

**Figure 3:**
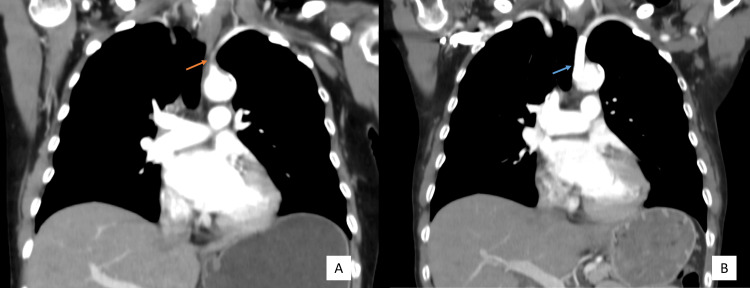
Computed tomography with angiogram showing subclavian stenosis before and after vascular intervention (A) Before, orange arrow indicating left subclavian stenosis. (B) After, blue arrow indicating vascular conduit in the left subclavian artery origin.

After more careful examination, including measuring the blood pressure in the four members, an interarm systolic pressure gradient of 59 mmHg was found, with an ankle-brachial index of 0.83 on the right and 0.86 on the left (Table 1). Additional tests were ordered. A neck and chest CTA revealed diffuse atherosclerotic disease, purely calcified or mixed component, involving: 1. thoracic artery; 2. aortic arch; 3. supra-aortic trunks; 3. brachiocephalic trunk origin; 4. coronary arteries; 5. left main carotid artery origin (50%-60%); 6. left subclavian artery with significant stenosis (Figure 3A). The arterial ultrasound of the right leg, where the patient had developed intermittent claudication (Fointaine Classification IIa/b), revealed hemodynamically significant stenosis of the main femoral artery.

**Table 1:** Highest systolic blood pressure measured in the four limbs with respect to ankle brachial index and interarm gradient * normal values of 1.00-1.40, being under 0.90 diagnostic of peripheral arterial disease [4]; ** normal values under 10 mmHg, being diagnostic of subclavian or axillary arterial stenosis values greater than 20 mmHg [4]. Note: Ankle-brachial index was calculated with the right arm systolic blood pressure.

	Systolic Blood Presure	Ankle-Brachial Index	Interarm gradient
Left Leg	119 mmHg	0.86*	
Right Leg	115 mmHg	0.83*	
Left arm	80 mmHg		59 mmHg**
Right arm	139 mmHg		

The patient was then referred to vascular surgery for her arterial disease, and continued treatment with high-potency statin and single antiplatelet therapy. She managed to achieve complete smoking cessation, something she was already seeking before the stroke, and her lipidic profile was improved (Table 2). 

**Table 2:** Lipidic profile before and after atorvastatin 40mg od and lifestyle modification advice, with difference at three months in percentage HDL: high-density lipoprotein; LDL: low-density lipoprotein

	Before	After	Difference at 3 months
Total cholesterol	193 mg/dL	112 mg/dL	-42%
HDL cholesterol	25 mg/dL	32 mg/dL	+28%
LDL cholesterol	120 mg/dL	60 mg/dL	-50%
Triglycerides	160 mg/dL	99 mg/dL	-38%

By the time of the vascular surgery’ appointment, she already had pain at rest on her right leg, with a preserved femoral pulse but no distal pulses. She was shortly submitted to an angioplasty of the right superficial femoral artery. Further ahead, due to persistent symptoms of cooling and paraesthesia in her left hand, she was submitted to percutaneous revascularization with stenting of the left subclavian artery (Figure 3B).

Carotid disease was not treated, since the stenosis was less than 70% and doubts have been raised about its contribution to the stroke (*versus* small vessels atherosclerosis). Given that the event occurred three months ago, it was decided not to treat mechanically and opt for medical therapy only.

Coronary disease, on the other hand, is still being managed, waiting for specific complementary tests to further stratify the disease level. Nonetheless, the patient is asymptomatic, with no reports of chest pain.

Education for the disease was also done during internal medicine appointments. Cardiovascular risk factors were addressed with encouragement on keeping smoking cessation, improving eating habits, in order to prevent diabetes and arterial hypertension, and keeping a moderate physical activity. The signs and symptoms that should motivate a visit to the emergency department were also explained, such as extremities pain at rest, increasing pain with less effort, persistent extremities colling or paresthesia, chest pain, as well as neurological deficits as she experienced before. The importance of therapeutic compliance was stressed. Multidisciplinary management is still in course by vascular surgery and internal medicine, with clinical monitoring and cardiovascular risk factors control.

## Discussion

In this case, we present a clinical case of a stroke that has revealed itself as being seldom a presentation of an undercover severe diffuse atherosclerotic disease. In fact, this patient has a polyvascular disease, defined as affecting two or more arterial beds [5].

Polyvascular disease can include any combination of PAD, cerebrovascular disease (CVD) and coronary artery disease (CAD). It has a worsened prognosis than either of them alone in terms of major adverse cardiovascular events (HR 1.99; 95% CI, 1.78-2.24; P < 0.001) and mortality (4.6% vs 2.4%) as shown in “Reduction of Atherothrombosis for Continued Health Registry” (REACH) at four-year follow-up, an international prospective registry including patients ≥ 45 years of age with established CAD, CVD, PAD, or ≥ 3 risk factors for atherosclerotic disease [5,6]. Interestingly, the worsened prognosis was verified despite polyvascular disease patients being more intensively treated. However, they were older, more often former or current smokers, and suffered more often from hypertension, diabetes, and diabetic nephropathy, which can contribute to the overall prognosis [6]. The indications for polyvascular disease screening, even in the presence of evidence of atherosclerotic disease in one territory are not defined [5,7].

Indeed, the management of these comorbid patients must be multisystemic, being time to intervention an important prognostic factor and determinant of morbidity and mortality [3]. A systemic review concerning delays in the management of chronic limb-threatening ischemia and diabetic foot ulceration, showed increased limb amputation, decreased wound healing, and increased mortality [3]. The causes identified varied from symptoms recognition to medical care seeking, specialty referral, to effective treatment. Multidisciplinary teams were pointed as an option to increase recognition and improve care. Case by case decisions considering comorbidities and global health status are recommended, particularly in polyvascular disease, where the internist, should have a central role [3,4,8].

 In our patient, cardiovascular risk was approached in a multidisciplinary way, resulting in the prevention of acute peripheric ischemia episodes and avoiding a possible amputation. Risk factors were partially controlled with smoking cessation and lowering LDL cholesterol by 50%. No other modifiable risks factors were found. However, further study (specially directed to CAD) and continuous effort to control dyslipidemia is still in course. Continuous awareness for the disease and optimizing its medical treatment is essential to prevent not only limb ischemia, but also major cardiovascular events.

## Conclusions

In our opinion, this clinical case demonstrates a severe disseminated arterial disease that could go underdiagnosis, with severe consequences, if we did not go further in our routine investigation of a stroke. A timely recognition, diagnosis, and referral were also crucial to the outcome. As internal medicine practitioners, it is our job to look at the patient in all its complexity and not only at the main problem. Only with multidisciplinary management, we can diminish cardiovascular burden.
